# A nomogram model for predicting intramyocardial hemorrhage post-PCI based on SYNTAX score and clinical features

**DOI:** 10.1186/s12872-024-03847-6

**Published:** 2024-03-25

**Authors:** Yin-shuang Yang, De-yang Xi, Yang Duan, Miao Yu, Kai Liu, Yan-kai Meng, Chun-feng Hu, Shu-guang Han, Kai Xu

**Affiliations:** 1 Xuzhou Medical University, Jiangsu, 221004 China; 2 Department of Cardiac Care Unit, The Affiliated Hospital of Xuzhou Medical University, Jiangsu, 221006 China; 3 Department of Radiology, The Affiliated Hospital of Xuzhou Medical University, Jiangsu, 221006 China

**Keywords:** Acute myocardial infarction, Cardiac magnetic resonance, SYNTAX score, Nomogram

## Abstract

**Objective:**

The aim of this study is to develop a nomogram model for predicting the occurrence of intramyocardial hemorrhage (IMH) in patients with Acute Myocardial Infarction (AMI) following Percutaneous Coronary Intervention (PCI). The model is constructed utilizing clinical data and the SYNTAX Score (SS), and its predictive value is thoroughly evaluated.

**Methods:**

A retrospective study was conducted, including 216 patients with AMI who underwent Cardiac Magnetic Resonance (CMR) within a week post-PCI. Clinical data were collected for all patients, and their SS were calculated based on coronary angiography results. Based on the presence or absence of IMH as indicated by CMR, patients were categorized into two groups: the IMH group (109 patients) and the non-IMH group (107 patients). The patients were randomly divided in a 7:3 ratio into a training set (151 patients) and a validation set (65 patients). A nomogram model was constructed using univariate and multivariate logistic regression analyses. The predictive capability of the model was assessed using Receiver Operating Characteristic (ROC) curve analysis, comparing the predictive value based on the area under the ROC curve (AUC).

**Results:**

In the training set, IMH post-PCI was observed in 78 AMI patients on CMR, while 73 did not show IMH. Variables with a significance level of *P* < 0.05 were screened using univariate logistic regression analysis. Twelve indicators were selected for multivariate logistic regression analysis: heart rate, diastolic blood pressure, ST segment elevation on electrocardiogram, culprit vessel, symptom onset to reperfusion time, C-reactive protein, aspartate aminotransferase, lactate dehydrogenase, creatine kinase, creatine kinase-MB, high-sensitivity troponin T (HS-TnT), and SYNTAX Score. Based on multivariate logistic regression results, two independent predictive factors were identified: HS-TnT (Odds Ratio [OR] = 1.61, 95% Confidence Interval [CI]: 1.21–2.25, *P* = 0.003) and SS (OR = 2.54, 95% CI: 1.42–4.90, *P* = 0.003). Consequently, a nomogram model was constructed based on these findings. The AUC of the nomogram model in the training set was 0.893 (95% CI: 0.840–0.946), and in the validation set, it was 0.910 (95% CI: 0.823–0.970). Good consistency and accuracy of the model were demonstrated by calibration and decision curve analysis.

**Conclusion:**

The nomogram model, constructed utilizing HS-TnT and SS, demonstrates accurate predictive capability for the risk of IMH post-PCI in patients with AMI. This model offers significant guidance and theoretical support for the clinical diagnosis and treatment of these patients.

## Introduction

Acute Myocardial Infarction (AMI) results from the rupture or erosion of atherosclerotic plaques in coronary arteries, causing acute constriction or obstruction of vessels and leading to sudden myocardial ischemia and necrosis [1, 2]. AMI, encompassing ST-segment elevation myocardial infarction (STEMI) and non-ST-segment elevation myocardial infarction (NSTEMI), is a critical cardiac event. STEMI may also present initially with acute heart failure [3]. Globally, ischemic heart disease stands as the foremost burden on health, as measured by disability-adjusted life years [4, 5]. Currently, Percutaneous Coronary Intervention (PCI), as endorsed by guidelines, has contributed to a reduction in mortality among AMI patients [6]. Nevertheless, despite effective epicardial reperfusion, a substantial number of AMI patients experience chronic heart failure due to poor microvascular function and inadequate recovery of myocardial perfusion, known as ‘no-reflow’ or microvascular obstruction (MVO) [7]. MVO can result in elevated intramyocardial pressure, leading to capillary rupture and Intramyocardial Hemorrhage (IMH) [8]. This hemorrhage further compromises myocardial tissue, exacerbating myocardial injury [9]. The ESC’s fourth universal definition of myocardial infarction guidelines provides a detailed categorization of myocardial infarction types based on underlying causes. This classification aids in tailoring treatment approaches according to the specific type of myocardial infarction, which can significantly impact patient prognosis [10].

Cardiac Magnetic Resonance (CMR), acknowledged as the gold standard for non-invasive cardiac structure and function assessment [11], can identify MVO and IMH using delayed gadolinium enhancement and T2* sequences, respectively [12]. The SYNTAX Score (SS) serves as a method to gauge the severity of coronary artery disease [13]. SS quantitatively assesses the complexity of coronary artery lesions based on anatomical characteristics such as lesion location, severity, morphology, bifurcation, and vascular calcification, effectively predicting the occurrence of adverse cardiovascular and cerebrovascular events. Studies have established a correlation between a higher SS, the extent of myocardial injury, and the Myocardial Salvage Index (MSI) post-PCI in AMI patients [14]. A higher SS is associated with a larger myocardial infarction (MI) area and a lower MSI, potentially indicating more severe myocardial reperfusion hemorrhage and unfavorable cardiac remodeling [15]. SS is an independent predictor of major adverse cardiac events (MACE) and three-year mortality post-direct PCI [16, 17]. Currently, several risk models and scoring systems have been developed to predict bleeding events following percutaneous coronary intervention (PCI) [18–20], such as the PRECISE-DAPT score. These models primarily focus on the bleeding risk in patients on antiplatelet therapy post-PCI, particularly significant bleeding events. However, no studies have yet explored the risk of intramyocardial hemorrhage in patients with myocardial infarction after PCI. Besides, the utilization of SS in predicting the occurrence of IMH post-PCI in AMI patients has not been reported to date. This study aims to construct a nomogram model using clinical data and SS to predict the occurrence of IMH in patients with AMI after PCI.

## Methods

### Study population

This retrospective study enrolled 216 patients with AMI who underwent emergency PCI at the Affiliated Hospital of Xuzhou Medical University between September 2019 and June 2023, and were randomly divided into a training set and a validation set at a ratio of 7:3. The sample size met the principle of 10 Events Per Variable (EPV). Inclusion criteria were: (1) AMI diagnosis following the European Society of Cardiology guidelines [6]; (2) First-time myocardial infarction patients with confirmed Infarct-Related Artery (IRA) through Coronary Angiography (CAG) post Percutaneous Transluminal Coronary Angioplasty (PTCA) who successfully underwent PCI; (3) Adequate angiographic image quality for SYNTAX Score (SS) calculation; (4) Underwent Cardiac Magnetic Resonance (CMR) within one week post-surgery with satisfactory image quality. Exclusion criteria were: (1) History of myocardial infarction; (2) Previous PCI or coronary artery bypass grafting; (3) Contraindications for late gadolinium enhancement (LGE)-CMR or coronary angiography (e.g., claustrophobia, contrast agent allergy); (4) Severe liver or kidney dysfunction; (5) Severe coagulation disorders or hemorrhagic diseases.

### Observation indicators

Patient data, including general and clinical information, were collected. Venous blood was drawn upon admission to measure initial myocardial enzyme levels, including Lactate Dehydrogenase, Creatine Kinase, Creatine Kinase-MB Isoenzyme, and High-Sensitivity Troponin T (HS-TnT). These myocardial enzyme levels were measured every 24 h, with the peak values of each indicator recorded. Additionally, N-terminal pro b-type natriuretic peptide (NT-proBNP) levels were assessed every 24 h, with the highest value being recorded as the peak NT-proBNP level. This study adheres to the principles of the 1975 Declaration of Helsinki and has received approval from the Ethics Committee of the Affiliated Hospital of Xuzhou Medical University (Approval No. XYFY2023-KL317-01).

### SYNTAX score calculation

The SYNTAX Score (SS) for each patient was retrospectively calculated based on the recommended SS algorithm. This involved scoring all coronary artery lesions with a diameter stenosis greater than 50% and vessels with a diameter larger than 1.5 mm [21]. The calculation was performed using the publicly accessible, web-based scoring calculator at http://www.syntaxscore.com.

### CMR examination and grouping criteria

CMR imaging was performed within the first week post-PCI using a 3.0-T scanner (Ingenia 3.0T, Philips, Netherlands). Patients were placed in a supine position, and images were obtained using a digital stream (dS) phased-array surface coil and an integrated dS posterior spinal matrix coil during breath-hold. After the administration of a gadolinium contrast agent (0.1 mmol/kg), cine images covering the left ventricular short axis (8–10 slices) were acquired. Late Gadolinium Enhancement (LGE) images were obtained 10–15 min after contrast administration. Imaging parameters included a slice thickness of 7 mm, echo time (TE) of 1.4 ms, repetition time (TR) of 2.8 ms, and a field of view (FoV) of 300 mm × 300 mm.

In Late Gadolinium Enhancement CMR (LGE-CMR), contrast agent washout is utilized to outline the areas of MVO. T2-weighted cardiac MRI highlights the tissue characteristics of myocardium and blood. On T2*-weighted CMR imaging, a low signal core within high signal edematous myocardium, identified as lesions with T2* values less than 20ms, is considered indicative of IMH. This appearance is attributed to the paramagnetic effects of hemoglobin degradation products [22]. Patients were categorized based on the presence or absence of IMH detected by CMR into groups with IMH and without IMH.

### Statistical methods

Statistical analyses in this study were performed using R software, version 4.2.2. For normally distributed quantitative data, means ± standard deviations were used for representation, with group comparisons conducted using the paired t-test. For non-normally distributed quantitative data, medians (interquartile range) were presented, and the Wilcoxon rank-sum test was applied for comparisons between groups. Categorical variables were compared between groups using the Chi-square test. Univariate and multivariate logistic regression analyses were employed to construct the nomogram model. The predictive ability of the model was assessed using ROC curve analysis, with the predictive value compared based on the area under the ROC curve. Calibration curves and decision curve analysis were generated to evaluate the consistency and accuracy of the model.

## Results

### Clinical data

**Fig. 1:**
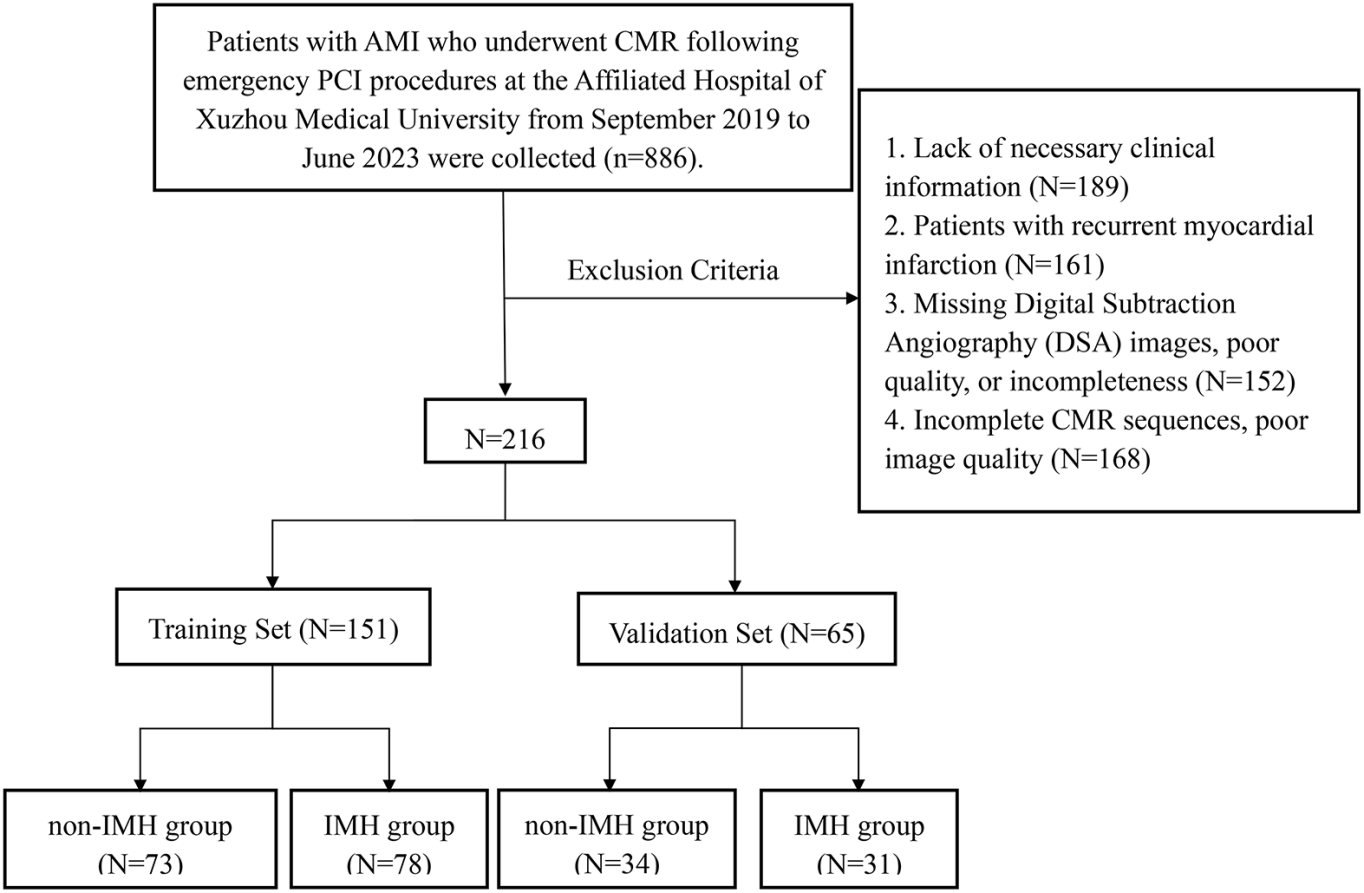
Flowchart

**Fig. 2:**
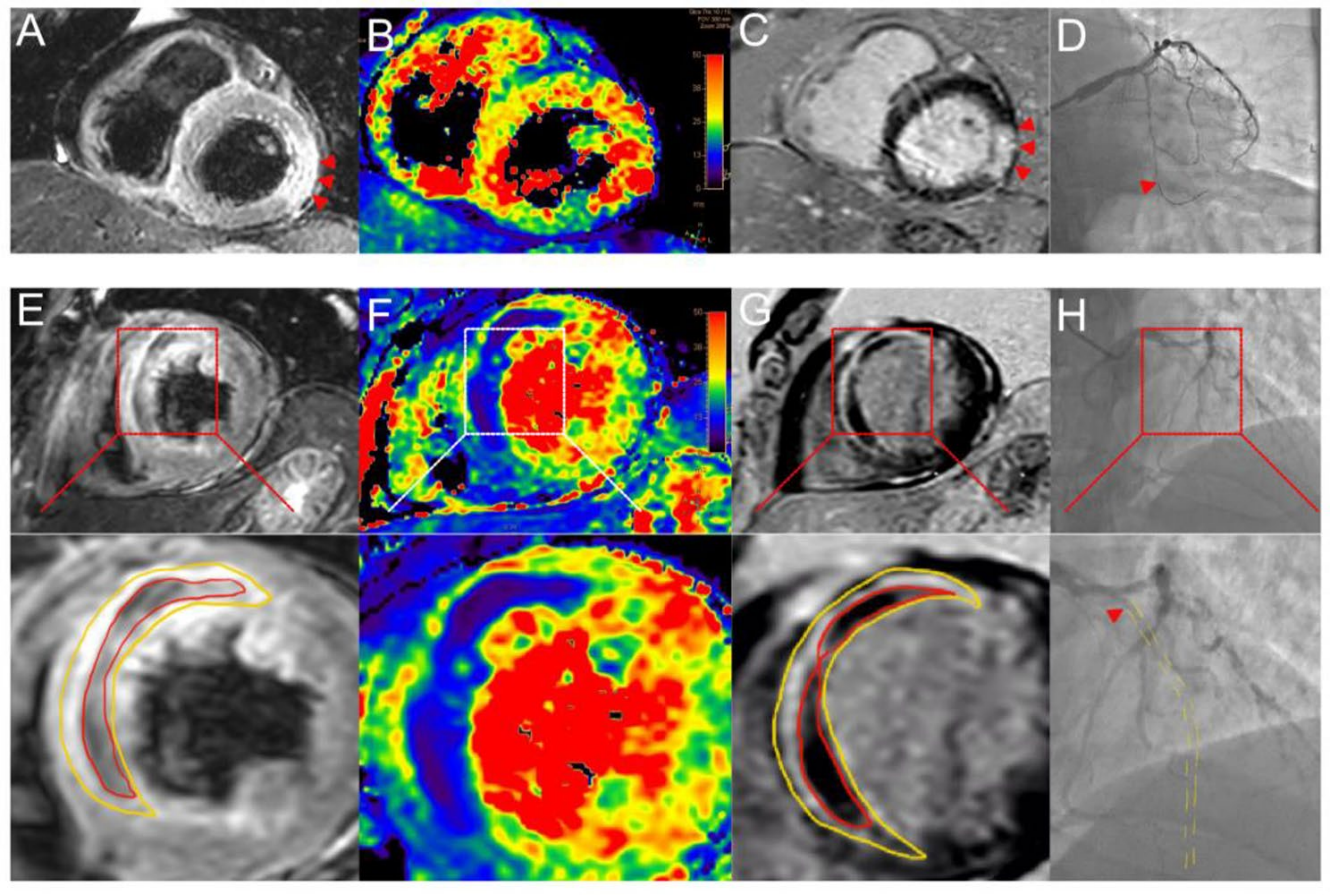
Two illustrative cases of CMR imaging and arteriography are presented. In the first case, without IMH (Panels A–D): (A) T2-weighted CMR reveals infarct zone hyperintensity (red arrowheads); (B) T2*-weighted CMR shows reduced infarct zone affinity; (C) LGE CMR highlights the extensive infarction (red arrowheads); (D) CAG reveals complete occlusion of the mid left circumflex artery (LCX). In the second case, featuring IMH (Panels E–H): (E) T2-weighted CMR identifies IMH as a hypointense core (red line circles) within a hyperintense zone (yellow line circles); (F) T2*-weighted CMR more clearly delineates the IMH, appearing as a dark area; (G) LGE CMR shows a hypointense core (red line circles) within a hyperintense zone (yellow line circles), indicative of microvascular injury; (H) CAG shows total occlusion of the left anterior descending artery (LAD)

The flowchart for the selection of AMI patients is depicted in Fig. 1. A total of 216 AMI patients were included in this study and randomly divided into a training set and a validation set in a 7:3 ratio. Based on the presence or absence of IMH as indicated by CMR, patients were classified into the IMH group (109 patients) and the non-IMH group (107 patients). Significant statistical differences were observed between the two groups in terms of heart rate, presence of ST-segment elevation myocardial infarction, culprit vessel, symptom onset to reperfusion time, C-reactive protein, aspartate aminotransferase, alanine aminotransferase, lactate dehydrogenase, creatine kinase, creatine kinase-MB isoenzyme, HS-TnT, NT-proBNP, and SS (Table 1). Representative examples of obtained CMR and arteriographic results are illustrated in Fig. 2. Table 2 presents a comparison of general and laboratory data between the training and validation sets, showing no statistical differences between the two groups.

**Table 1:** Comparison of general and clinical data between the two patient groups

	Non-IMH group	IMH group	Statistical value	P value
	*N* = 107	*N* = 109		
Age	54.9 ± 12.1	54.5 ± 11.8	*t* = 0.258	0.796
Gender			*X*^*2*^ = 0.201	0.654
Male	91 (85.0%)	95 (87.2%)		
Female	16 (15.0%)	14 (12.8%)		
BMI (kg/m^2^, x ± s)	25.5 ± 3.04	26.0 ± 3.22	*t*=-1.109	0.268
Heart rate	77.9 ± 11.9	81.7 ± 12.3	*t*=-2.320	0.021
Systolic blood pressure (mmHg)	125 [112;140]	120 [111;133]	*Z*= -1.342	0.180
Diastolic blood pressure (mmHg, x ± s)	79.4 ± 14.0	81.1 ± 13.4	*t*=-0.915	0.361
**Medical history**				
Hypertension (Classification)			*X*^*2*^ = 4.471	0.215
0	60 (56.1%)	57 (52.3%)		
1	9 (8.41%)	4 (3.67%)		
2	13 (12.1%)	11 (10.1%)		
3	25 (23.4%)	37 (33.9%)		
Smoking history			*X*^*2*^ = 0.690	0.406
No	46 (43.0%)	53 (48.6%)		
Yes	61 (57.0%)	56 (51.4%)		
Dyslipidemia			*X*^*2*^ = 0.155	0.693
No	59 (55.1%)	63 (57.8%)		
Yes	48 (44.9%)	46 (42.2%)		
Diabetes			*X*^*2*^ = 1.093	0.296
No	85 (79.4%)	80 (73.4%)		
Yes	22 (20.6%)	29 (26.6%)		
**History of drug use**				
Clopidogrel			*X*^*2*^ = 0.004	0.947
No	86 (80.4%)	88 (80.7%)		
Yes	21 (19.6%)	21 (19.3%)		
Beta-blocker			*X*^*2*^ = 0.690	0.406
No	61 (57.0%)	56 (51.4%)		
Yes	46 (43.0%)	53 (48.6%)		
ACEI/ARB			*X*^*2*^ = 0.323	0.569
No	63 (58.9%)	60 (55.0%)		
Yes	44 (41.1%)	49 (45.0%)		
**Clinical data**				
Killip classification			*X*^*2*^ < 0.001	1.000
I	99 (92.5%)	101 (92.7%)		
II/III/IV	8 (7.48%)	8 (7.34%)		
ECG			*X*^*2*^ = 5.925	0.015
STEMI	78 (72.9%)	94 (86.2%)		
NSTEMI	29 (27.1%)	15 (13.8%)		
Criminal vessel			*X*^*2*^ = 16.153	< 0.001
LAD	42 (39.3%)	71 (65.1%)		
LCX	19 (17.8%)	16 (14.7%)		
RCA	46 (43.0%)	22 (20.2%)		
Time from symptom onset to reperfusion (min)	360 [180;720]	240 [180;420]	*Z*= -2.35	0.019
GLU (mmol/L) ^*^	5.69 [5.01;6.99]	6.06 [5.05;8.32]	*Z*= -1.422	0.155
HbA1c (%)	5.90 [5.60;6.65]	5.90 [5.60;6.90]	*Z*= -0.387	0.699
CRP (mg/L)	19.5 [8.10;42.3]	39.1 [18.10;68.4]	*Z*= -3.945	< 0.001
PLT / LYM	89.1 [72.3;106]	87.0 [72.7;109]	*Z*= -0.016	0.987
AST (U/L)	119 [62.5;176]	311 [215;508]	*Z*= -8.294	< 0.001
ALT (U/L)	39.0 [29.0;52.5]	56.0 [39.0;75.0]	*Z*= -5.335	< 0.001
LDH (×10^2^U/L)	5.89 [3.42;8.31]	11.7 [7.64;19.50]	*Z*= -7.538	< 0.001
CK (×10^3^U/L)	1.00 [0.52;1.54]	3.84 [1.60;6.66]	*Z*= -8.247	< 0.001
CKMB (ng/ml)	72.2 [26.4;136]	254 [130;300]	*Z*= -7.336	< 0.001
HS-TnT (×10^3^ng/L)	1.90 [1.11;3.22]	6.72 [4.10;9.75]	*Z*= -9.372	< 0.001
NT-proBNP (×10^3^pg/L)	0.87 [0.47;1.44]	1.33 [0.70;2.10]	*Z*= -3.048	0.002
SS (×10)	1.80 [1.30;2.12]	2.65 [2.25;3.25]	*Z*= -7.568	< 0.001

**Table 2:** General and clinical data of patients in the training and validation sets

	Training set	Verification set	Statistical value	P value
	*N* = 151	*N* = 65		
Age	55.0 [47.0;64.0]	56.0 [46.0;64.0]	*Z*= -0.174	0.868
Gender			*X*^*2*^ < 0.001	1.000
Male	130 (86.1%)	56 (86.2%)		
Female	21 (13.9%)	9 (13.8%)		
BMI (kg/m^2^, x ± s)	25.7 [23.9;27.8]	25.0 [23.4;27.5]	*Z*= -1.238	0.190
Heart Rate	79.7 ± 12.5	80.0 ± 11.6	*t* = 0.207	0.837
Systolic blood pressure (mmHg)	124 [112;137]	123 [113;136]	*Z*= -1.342	0.996
Diastolic blood pressure (mmHg, x ± s)	80.1 ± 13.8	80.4 ± 13.5	*t* = 0.162	0.872
**Medical history**				
Hypertension (Classification)			*X*^*2*^ = 2.141	0.544
0	78 (51.7%)	39 (60.0%)		
1	11 (7.28%)	2 (3.08%)		
2	17 (11.3%)	7 (10.8%)		
3	45 (29.8%)	17 (26.2%)		
Smoking history			*X*^*2*^ = 0.004	1.000
No	69 (45.7%)	30 (46.2%)		
Yes	82 (54.3%)	35 (53.8%)		
Dyslipidemia			*X*^*2*^ = 0.468	0.494
No	83 (55.0%)	39 (60.0%)		
Yes	68 (45.0%)	26 (40.0%)		
Diabetes			*X*^*2*^ = 0.221	0.638
No	114 (75.5%)	51 (78.5%)		
Yes	37 (24.5%)	14 (21.5%)		
**History of drug use**				
Clopidogrel			*X*^*2*^ = 0.783	0.376
No	124 (82.1%)	50 (76.9%)		
Yes	27 (17.9%)	15 (23.1%)		
Beta-blocker			*X*^*2*^ = 0.056	0.814
No	81 (53.6%)	36 (55.4%)		
Yes	70 (46.4%)	29 (44.6%)		
ACEI/ARB			*X*^*2*^ = 0.354	0.552
No	84 (55.6%)	39 (60.0%)		
Yes	67 (44.4%)	26 (40.0%)		
**Clinical data**				
Killip classification			*X*^*2*^ < 0.001	1.000
I	140 (92.7%)	60 (92.3%)		
II/III/IV	11 (7.28%)	5 (7.69%)		
ECG			*X*^*2*^ = 1.917	0.166
STEMI	124 (82.1%)	48 (73.8%)		
NSTEMI	27 (17.9%)	17 (26.2%)		
Criminal vessel			*X*^*2*^ = 5.225	0.073
LAD	86 (57.0%)	27 (41.5%)		
LCX	20 (13.2%)	15 (23.1%)		
RCA	45 (29.8%)	23 (35.4%)		
Time from symptom onset to reperfusion (min)	300 [180;540]	300 [210;720]	*Z*= -2.350	0.208
**GLU (mmol/L)** ^*^	5.89 [5.10;7.32]	5.71 [4.94;7.09]	*Z*= -1.422	0.246
HbA1c (%)	6.00 [5.70;6.90]	5.80 [5.60;6.50]	*Z*= -0.387	0.115
CRP (mg/L)	27.5 [11.7;60.6]	27.6 [10.6;60.5]	*Z*= -3.945	0.974
PLT / LYM	88.3 [70.2;106]	88.1 [75.2;108]	*Z*= -0.016	0.562
AST (U/L)	191 [99.5;330]	191 [100;408]	*Z*= -8.294	0.917
ALT (U/L)	45.0 [33.0;61.5]	51.0 [35.0;68.0]	*Z*= -5.335	0.488
LDH (×10^2^U/L)	7.84 [4.99;12.0]	8.93 [4.60;14.1]	*Z*= -7.538	0.428
CK (×10^3^U/L)	1.53 [0.78;3.93]	1.60 [0.97;3.70]	*Z*= -8.247	0.812
CKMB (ng/ml)	124 [53.8;272]	189 [64.3;264]	*Z*= -7.336	0.646
HS-TnT (×10^3^ng/L)	3.59 [1.68;6.88]	3.75 [1.75;7.29]	*Z*= -9.372	0.981
NT-proBNP (×10^3^pg/L)	1.12 [0.65;1.89]	1.01 [0.45;1.91]	*Z*= -3.048	0.294
SS (×10) ^*^	2.25 [1.50;2.85]	2.15 [1.60;2.80]	*Z*= -7.568	0.759

### Univariate and multivariate logistic regression analysis of independent predictors for IMH post-PCI in AMI patients

In Table 3, the results of the univariate logistic regression analysis showed significant statistical differences between the two groups in baseline heart rate, diastolic blood pressure, ST-segment elevation, culprit vessel, symptom onset to reperfusion time, C-reactive protein, aspartate aminotransferase, myocardial enzymes, troponin, and SS. Variables with *P* < 0.05 in the univariate analysis were included in the multivariate logistic regression analysis. The results identified HS-TnT and SS as independent risk factors for the occurrence of IMH post-PCI in AMI patients (both P values < 0.05), with cut-off values of 3.61 and 2.23, respectively. Table 3 presents the OR, 95% confidence intervals, and P values for both the univariate and multivariate logistic regression analyses.

**Table 3:** Logistic regression analysis of independent predictors for IMH post-PCI in AMI patients

Clinical factors	Univariate logistic regression			Multivariate logistic regression		
	OR	95%CI	P	OR	95%CI	P
Age	0.99	0.97–1.02	0.716			
Gender	1.21	0.48–3.04	0.690			
BMI	1.07	0.96–1.19	0.199			
Heat rate	1.03	1.01–1.06	0.016	1.01	0.97–1.06	0.524
Systolic blood pressure	1.00	0.99–1.02	0.816			
Diastolic blood pressure	1.02	1.00-1.05	0.047	1.02	0.98–1.06	0.379
Hypertension (Classification): 0 VS 1	0.39	0.10–1.60	0.193			
Hypertension (Classification): 0 VS 2	0.94	0.33–2.68	0.901			
Hypertension (Classification): 0 VS 3	1.91	0.90–4.06	0.093			
Smoking history	0.56	0.29–1.07	0.081			
Hyperlipidemia	0.89	0.47–1.68	0.713			
Diabetes history	1.76	0.82–3.77	0.144			
Clopidogrel	1.01	0.44–2.32	0.982			
Beta-blocker	1.35	0.71–2.58	0.354			
ACEI/ARB	1.29	0.68–2.46	0.434			
Killip classification	0.76	0.22–2.62	0.670			
ECG	0.32	0.13–0.80	0.014	0.59	0.13–2.54	0.485
Criminal vessel: LAD VS LCX	0.44	0.16–1.18	0.101	2.39	0.61–9.86	0.216
Criminal vessel: LAD VS RCA	0.44	0.21–0.91	0.027	0.73	0.23–2.27	0.585
Time from symptom onset to reperfusion (min)	1.00	1.00–1.00	0.010	1.00	1.00–1.00	0.344
GLU(mmol/L) ^*^	1.10	0.99–1.22	0.071			
HbA1c	1.08	0.89–1.30	0.432			
CRP	1.01	1.00-1.02	0.039	1.01	1.00-1.02	0.221
PLT / LYM	1.00	0.99–1.01	0.739			
AST	1.01	1.00-1.01	< 0.001	0.99	0.99-1.00	0.193
ALT	1.00	1.00–1.00	0.739			
LDH	1.23	1.13–1.34	< 0.001	1.03	0.90–1.20	0.651
CK	2.04	1.56–2.68	< 0.001	1.52	1.00-2.51	0.078
CKMB	1.01	1.01–1.02	< 0.001	1.00	0.99–1.01	0.759
HS-TnT	1.87	1.53–2.28	< 0.001	1.61	1.21–2.25	0.003
NT-proBNP	1.23	0.98–1.54	0.069			
SS	4.26	2.53–7.18	< 0.001	2.54	1.42–4.90	0.003

### Construction of the nomogram

**Fig. 3:**
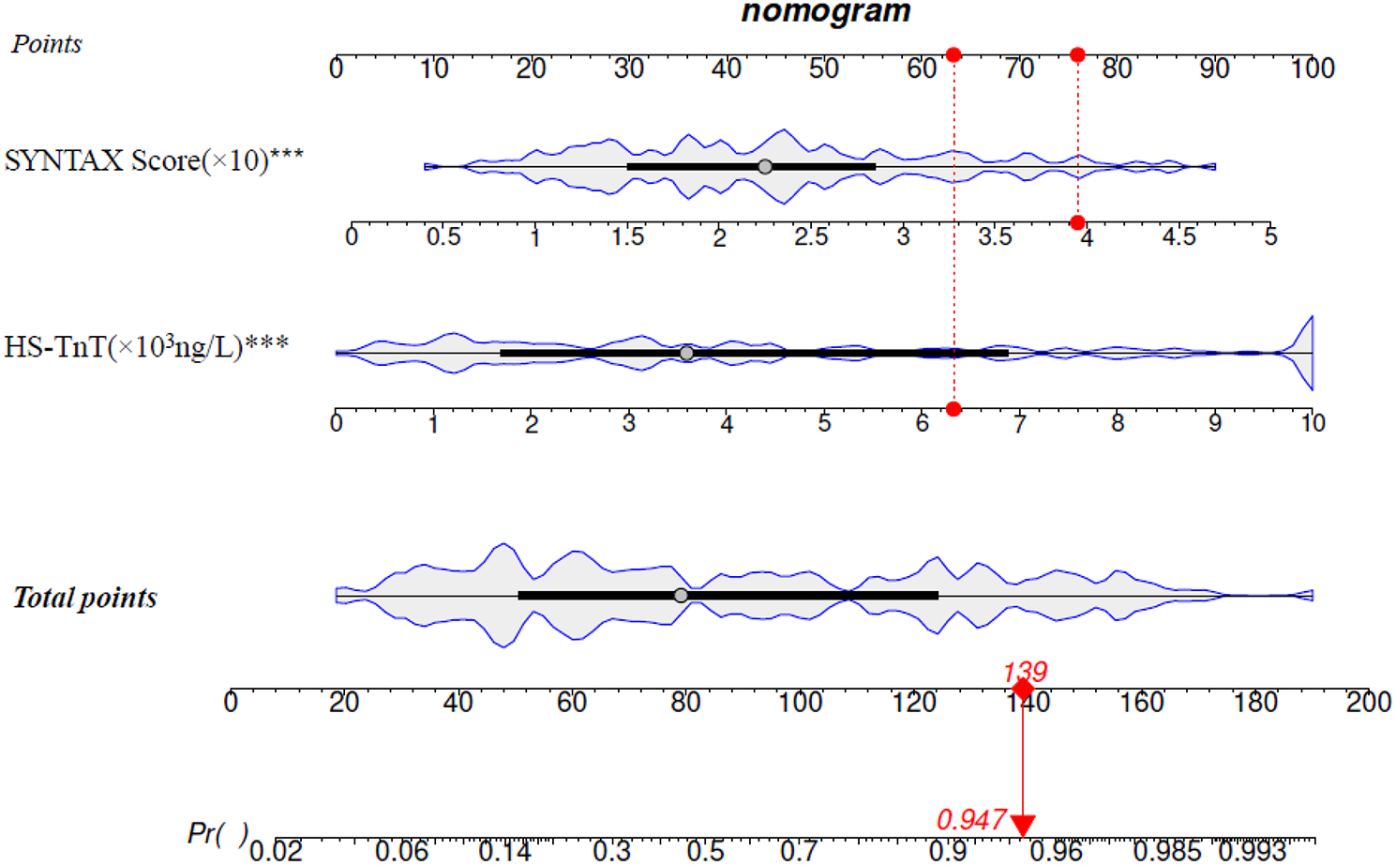
Construction of nomogram for predicting the occurrence of IMH post-PCI in AMI patients

To better address clinical needs, a predictive model for assessing the occurrence of IMH post-PCI in patients with AMI was developed. This model was based on the independent predictors identified through multivariate logistic regression and their corresponding coefficients. The formula of the model is: log(p/(1-p)) = -4.22 + 0.52HS-TnT + 0.97SS. A nomogram was then created based on this predictive model (Fig. 3). Using this formula, we can accurately calculate the risk of IMH in patients with AMI post- PCI based on their HS-TnT and SS.

### Accuracy assessment of the nomogram

**Fig. 4:**
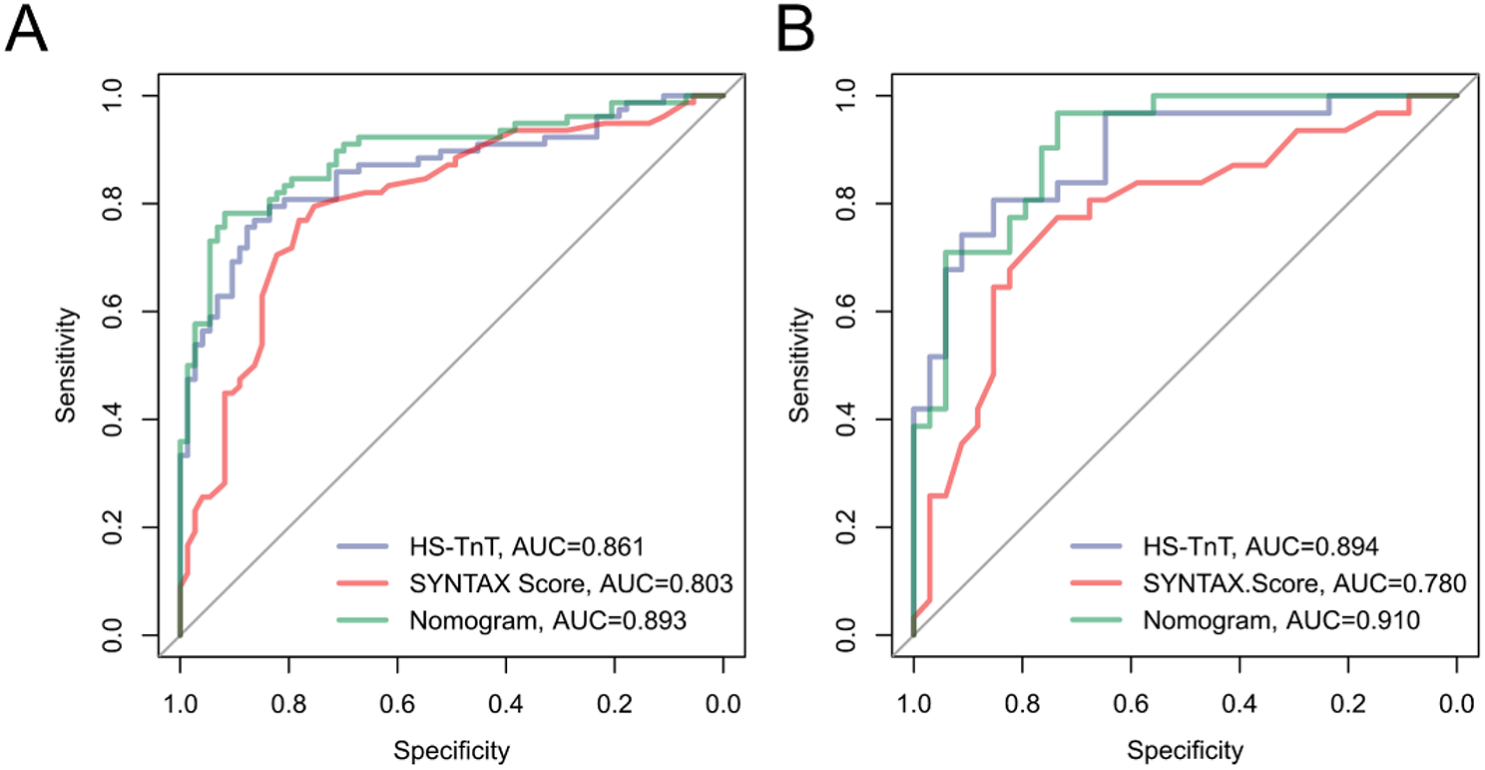
ROC curves of the nomogram model on the training set (A) and validation set (B)

**Fig. 5:**
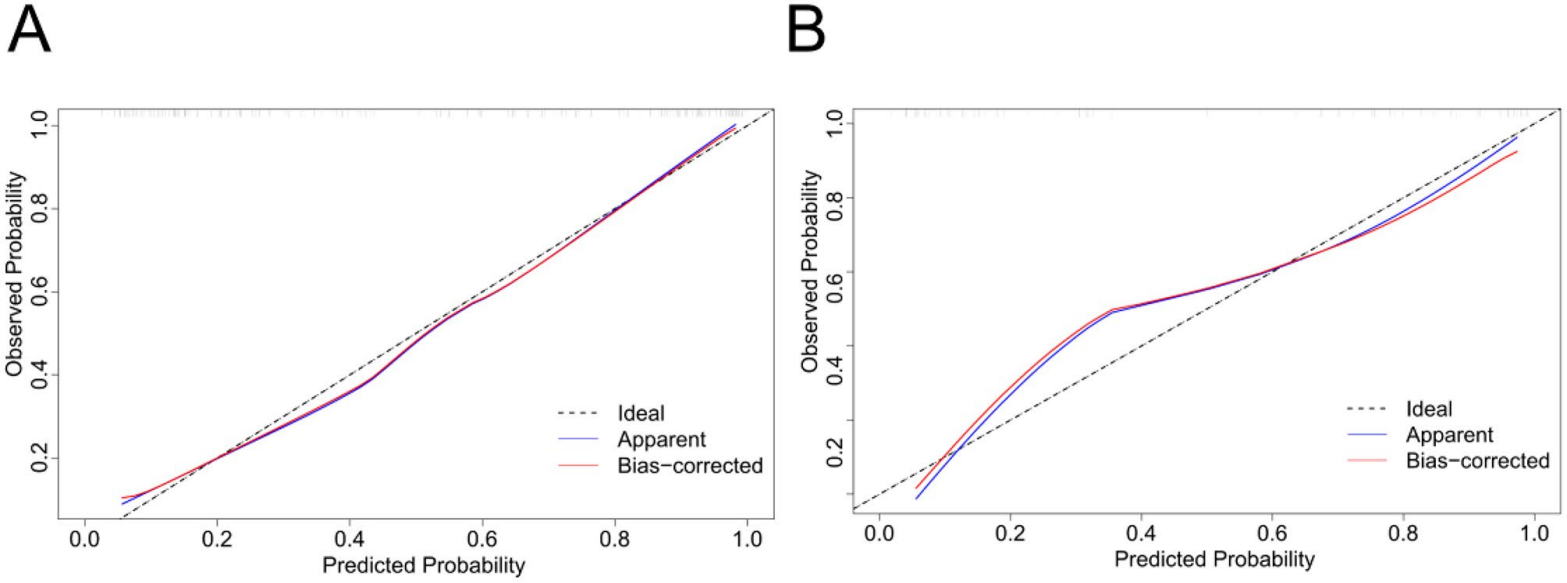
Calibration curves of the nomogram model on the training set (A) and validation set (B)

**Fig. 6:**
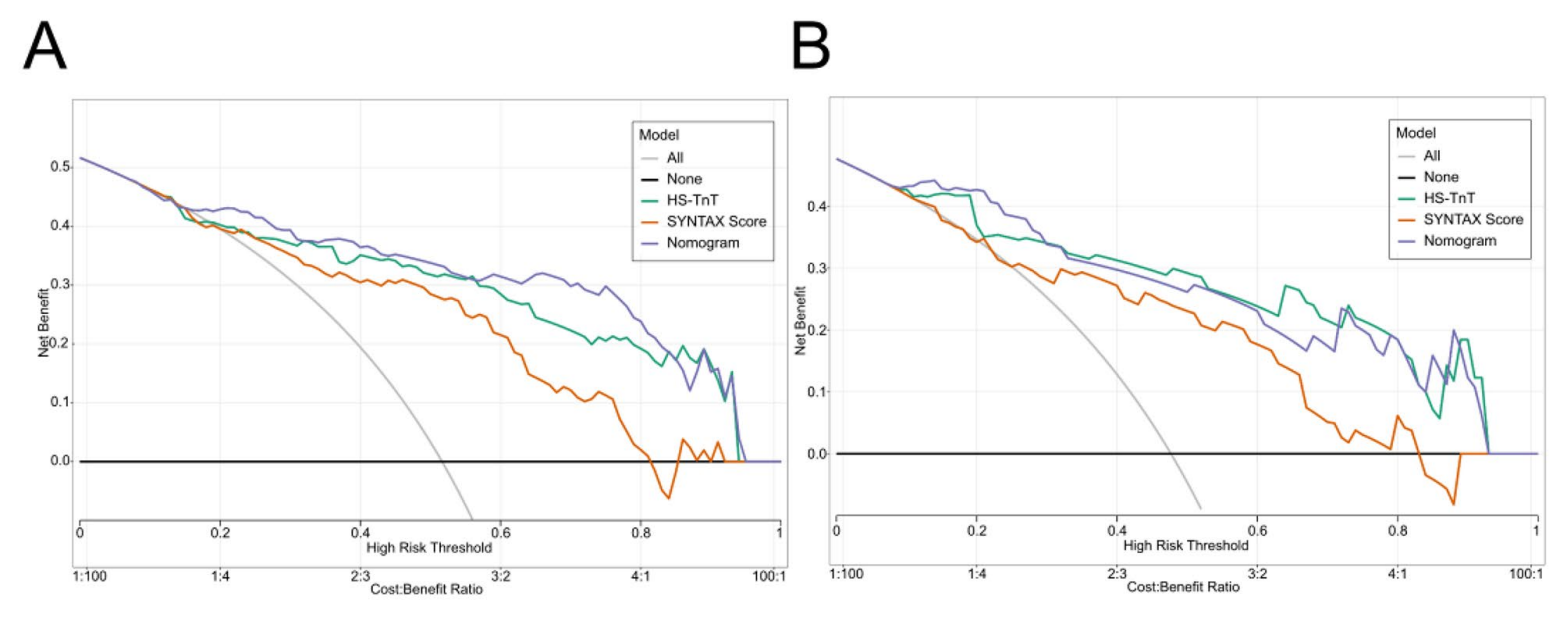
Decision curves of the nomogram model on the training set (A) and validation set (B)

To verify the accuracy of the constructed nomogram model, ROC curves were plotted. In the training set, the AUC of the nomogram model was 0.893 (95% CI: 0.840–0.946). In the validation set, the AUC was 0.910 (95% CI: 0.823–0.970), indicating that the model exhibits good accuracy in both the training and validation sets (Fig. 4). Additionally, calibration curves were drawn for both the training and validation sets (Fig. 5) to assess the model’s accuracy. The results showed that the predicted probabilities of the nomogram closely match the actual probabilities, further indicating the model’s reliability. The Hosmer-Lemeshow goodness-of-fit test showed no significant difference (X^2^ = 3.68, *P* = 0.885), affirming the model’s fit. Finally, decision curve analysis was used to compare the predictive capabilities of the nomogram with the independent risk factors. The decision curve indicated that the nomogram model’s predictions for post-PCI IMH in AMI patients were superior to those based on independent risk factors alone (Fig. 6).

## Discussion

AMI represents the most severe pathophysiological state of coronary heart disease, posing an immediate threat to patient survival [23]. Presently, PCI combined with antithrombotic therapy is the primary method for re-establishing coronary circulation in AMI patients [24]. However, some patients experience myocardial hemorrhage due to severe ischemic injury to the myocardium during reperfusion and MVO post-PCI, leading to vascular integrity disruption and extravasation of red blood cells [25]. In a clinical study conducted by Noé Corpataux et al. [26] on AMI patients post-PCI, the overall incidence of IMH was found to be as high as 43%. Therefore, accurate prediction of IMH post-PCI is of significant importance for the prognosis of AMI patients.

CMR is a useful non-invasive technique for determining the presence of MVO and IMH)in patients following reperfused AMI. Currently, dark-blood T2*-based CMR and bright-blood T2*-weighted CMR are becoming interchangeably used for the detection of IMH, enabling effective diagnosis of IMH [27]. Interestingly, studies have shown that positron emission tomography/magnetic resonance imaging (PET/MRI) is gradually providing new insights into the molecular mechanisms of the healing process post-myocardial infarction. In the context of myocardial infarction, PET/MRI can provide important biomarker information that reveals key processes such as myocardial metabolic status, inflammatory responses, apoptosis, and angiogenesis during the repair process [28]. Through this multimodal imaging approach, researchers and clinicians can gain a comprehensive view of the healing process after myocardial infarction, which is of significant importance for optimizing treatment strategies, assessing therapeutic effects, and predicting patient prognosis.

The Syntax Score (SS) is an established tool for assessing the complexity of coronary artery disease (CAD), particularly in patients with multivessel coronary artery disease and/or left main coronary artery lesions [29, 30]. The SS is primarily based on the location, type, and number of coronary artery lesions, providing robust theoretical support for the choice between PCI and Coronary Artery Bypass Grafting (CABG) [31, 32]. Studies have demonstrated that SS can predict the prognosis of patients undergoing PCI or CABG, offering a significant theoretical basis for evaluating the severity and prognosis of coronary heart disease [33]. High-sensitivity Troponin T (HS-TnT) is a highly specific biomarker for myocardial injury assessment [34]. Troponin testing, as the preferred and essential condition for diagnosing myocardial infarction, reduces the missed diagnosis rate of acute coronary syndrome and myocardial infarction and is widely used in clinical settings [35, 36]. Our study retrospectively analyzed the clinical indicator levels in patients with AMI post-PCI and calculated the corresponding SS based on coronary angiography, using Cardiac Magnetic Resonance (CMR) as the gold standard for grouping [37]. We found that HS-TnT levels and SS are independent risk factors for Intramyocardial Hemorrhage (IMH) post-PCI in AMI patients. Studies indicate that reperfusion can lead to necrosis within the epicardial wavefront, thereby elevating HS-TnT levels. We believe that compared to traditional troponins, HS-TnT offers higher sensitivity and specificity, allowing for earlier and more accurate detection of myocardial cell damage, which is closely related to intramyocardial hemorrhage. In this study, we also observed that patients with increased SS are more prone to intramyocardial hemorrhage. This susceptibility may be linked to the heavier burden of coronary atherosclerosis in patients with higher SS, where the revascularization and stent placement lead to mechanical disruption of the compromised vessels. A predictive model for IMH was constructed based on HS-TnT levels and SS. The results indicated that patients with IMH had higher HS-TnT levels and SS compared to those without IMH (Odds Ratio = 1.61/2.54, 95% Confidence Interval: 1.21–2.25/1.42–4.90, *P* = 0.003/0.003), consistent with findings by Liu et al. that troponin peak levels post-direct PCI are higher in patients with bleeding (*P* < 0.01) [38]. Although the AUC value of SS in ROC analysis is not as significant as HS-TnT levels, combining these two indicators through a nomogram model yields a predictive power greater than their independent predictive values. By calculating the scores of AMI patients post-PCI using the nomogram model, we can identify patients at high risk of IMH early, allowing for timely and appropriate clinical intervention, which is crucial for improving patient outcomes.

However, this study has several limitations: (1) It is a single-center retrospective study with a relatively small sample size. (2) There was no follow-up on patient outcomes and major adverse cardiac events (MACE), leading to a lack of systematic and comprehensive analysis of the prognosis of patients with IMH. (3) In this study, the assessment of SS was conducted by experienced professionals in a single center, and its predictive value in everyday clinical practice may require further evaluation through multicenter, double-blind studies.

## Conclusion

In summary, the nomogram model constructed using HS-TnT and SS can accurately predict the risk of IMH post-PCI in patients with AMI. This model can guide further diagnosis and treatment post-PCI in AMI patients, contributing to improved patient prognosis.

## Data Availability

The data analyzed in this study can be obtained from the corresponding authors upon request.

## Abbreviations

ECG: Electrocardiogram
STEMI: ST-Segment Elevation Myocardial Infarction
NSTEMI: Non-ST-Segment Elevation Myocardial Infarction
LAD: Left Anterior Descending artery
LCX: Left Circumflex artery
RCA: Right Coronary Artery
GLU: Glucose
HbA1c: Hemoglobin A1c
CRP: C-Reactive Protein
PLT: Platelets
LYM: Lymphocytes
AST: Aspartate Aminotransferase
ALT: Alanine Aminotransferase
LDH: Lactate Dehydrogenase
CK: Creatine Kinase
CKMB: Creatine Kinase-MB
HS-TnT: High-Sensitivity Troponin T
NT-proBNP: N-Terminal pro b-type Natriuretic Peptide
SS: SYNTAX Score
